# Characterization of influenza A virus induced transposons reveals a subgroup of transposons likely possessing the regulatory role as eRNAs

**DOI:** 10.1038/s41598-022-06196-6

**Published:** 2022-02-09

**Authors:** Steven S. Shen, Hezkiel Nanda, Constantin Aliferis, Ryan A. Langlois

**Affiliations:** 1 Institute for Health Informatics, University of Minnesota, Minneapolis, MN 55455 USA; 2 Department of Microbiology and Immunology, Center for Immunology, University of Minnesota, Minneapolis, MN 55455 USA; 3 Clinical Translational Science Institute, University of Minnesota, Minneapolis, MN 55455 USA

**Keywords:** Virology, Virus-host interactions, Microbiology, Computational biology and bioinformatics, Gene regulatory networks

## Abstract

Although many studies have observed genome-wide host transposon expression alteration during viral infection, the mechanisms of induction and the impact on the host remain unclear. Utilizing recently published influenza A virus (IAV) time series data and ENCODE functional genomics data, we characterized virus induced host differentially expressed transposons (virus-induced-TE) by investigating genome-wide spatial and functional relevance between the virus-induced-TEs and epigenomic markers (e.g. histone modification and chromatin remodelers). We found that a significant fraction of virus-induced-TEs are derived from host enhancer regions, where CHD4 binding and/or H3K27ac occupancy is high or H3K9me3 occupancy is low. By overlapping virus-induced-TEs to human enhancer RNAs (eRNAs), we discovered that a proportion of virus-induced-TEs are either eRNAs or part of enhancer RNAs. Upon further analysis of the eRNA targeted genes, we found that the virus-induced-TE related eRNA targets are overrepresented in differentially expressed host genes of IAV infected samples. Our results suggest that changing chromatin accessibility from repressive to permissive in the transposon docked enhancer regions to regulate host downstream gene expression is potentially one of the virus and host cell interaction mechanisms, where transposons are likely important regulatory genomic elements. Our study provides a new insight into the mechanisms of virus-host interaction and may lead to novel strategies for prevention and therapeutics of IAV and other virus infectious diseases.

## Introduction

We recently demonstrated that host transposon element (TE) expression is largely impacted by viral infection^1^. The findings have been confirmed across multiple organisms, e.g. human, mouse, and drosophila^2^ and numerous viral species including HIV1^3^ and SARS-CoV-2^4^, which indicates that the host transposon expression alteration is a fundamental phenomenon of viral infectious diseases. Although hypotheses and speculation about possible roles of virus induced differentially expressed TEs (virus-induced-TEs) are emerging, understanding the mechanism of induction and impact of transposon expression alteration on the host remains largely unclear. A recent report shows that TEs are involved in chromatin looping and CTCF bindings^5^. In *Arabidopsis thaliana* plants, the DDM1 mutation causes significant reduction of DNA methylation in the TE loci, where Snf2 family member DDM1 as a chromatin remodeler plays an important role in suppression of TE transcription^6^. Igolkina, et al. also reported that the connection between TEs and histone markers may impact gene expression regulation^7^. These interesting findings are suggestive of possible gene expression regulation roles by TE transcripts, which prompted our investigation into the genomic spatial relationship between the virus-induced-TE and permissive and/or repressive epigenomic markers, such as histone markers, chromatin remodelers, and transcription factors.

Advances in genome-wide enhancer RNA studies^8,9^, including on histone occupancy/transcription factor binding sites (ENCODE project^10^) and computational algorithms for functional genomic element predictions^11^, facilitate a deep investigation into the virus-induced-TEs and their spatial relationship to the functional genomic elements. Taking advantage of our recently published studies on influenza A virus (IAV)^12^, we further characterized host virus-induced-TEs by examining their spatial relationship to known functional genomic elements such as chromatin histone markers. We have found that a significant number of virus-induced-TEs (~ 9%) are derived from the chromatin histone marker H3K27ac pre-occupied regions, of which a fraction are also chromatin remodeler CHD4 binding sites. In addition to the H3K27ac occupied genome regions, the chromatin histone marker H3K9me3 regions are also found to accommodate about 2.2% of virus-induced-TEs. In a parallel analysis, by querying a computational predicted human enhancer database with virus-induced-TEs, we discovered that about 9.6% of IAV-induced TEs overlap the predicted distal enhancers. Querying the same enhancer database with other viral datasets^13,14^, we revealed that about 7–9% of SARS-CoV-2-induced TEs overlap the predicted enhancers, with variation in different experiments; about 7.7–7.9% of RSV and 9.8% of HPIV3 induced TEs overlap the predicted enhancers, respectively.

To understand the potential functional role of virus-induced-TEs in the host, we carefully examined the genomic loci of IAV-induced-TEs to known enhancer RNAs (eRNAs). We did this due to the important regulatory roles eRNA plays in the genome for tissue specific gene expression regulation^15^^,^^16^^,^^17^. We found that ~ 9.2% of IAV-induced-TEs overlap eRNAs (TE eRNAs). Furthermore, we also found that about 44% of the TE eRNA targets (2144) are overrepresented in the differentially expressed gene list (4824 DE genes) of IAV infected samples, in which both upregulated and downregulated TE eRNA targets are significantly overrepresented in the upregulated DE genes (*p* < 0.05).

Taken together, our study is the first evidence of a connection between a subgroup of IAV-induced-TEs and host enhancer RNAs. Our results indicate that the enhancers docked by this group of IAV-induced-TEs are likely less permissive and/or poised prior to virus infection. However, such enhancers become more accessible and active during virus infection, resulting in host transposon expression changes and possible downstream gene expression alteration. Based on our data, we introduce a new virus-host interaction model, showing that viruses likely influence host gene expression by activating poised enhancers and promoting eRNA transcription, where host TEs may play complex roles in the virus host interaction and gene expression regulation. This model is based on IAV but may also apply to other RNA viruses including SARS-CoV-2, RSV and HPIV3. Our discovery provides new insight into the mechanisms of virus-host interaction and may lead to novel preventive and therapeutic targets for IAV and other RAN virus infection diseases.

### Host transposon elements expression alteration during early stages of IAV infection

**Figure 1:**
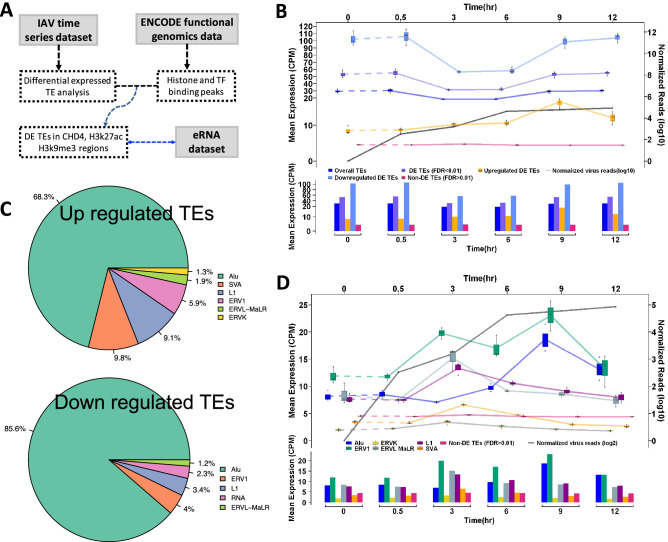
The temporal relationship between up regulated TEs or TE families and virus replication in the IAV early infection. (**A**) Outline of data sources and analysis strategy. (**B**) The dynamics of TE expression in the early stage of IAV infection. The upregulated DE TEs (yellow) correlates to virus replication (black) temporally, the non-DE TEs (pink) remain steady. (**C**) The top TE families in up (upper panel) and down (lower panel) regulated TEs. (**D**) The dynamics of top up regulated TE families at each time point in contrast to Non-DE TEs (pink).

All virus infection data used for this study was obtained from the public database (e.g. GSE147832 is an accession number of the NCBI GEO database). The data analysis scheme is highlighted in Fig. 1A. In this study, we first measured the overall transcriptome level for host transposons and genes in each virus infection time point in order to obtain a global picture of the dynamic relationship between the host transcriptome and the viral replication progression. The overall mean expression of 41,499 detectable TEs declined by ~ 38% between 3 and 6 h post-infection samples in comparison to the mock. The mean expression of overall TEs reduction is primarily reflected in the expression level changes of total differentially expressed TEs (DE TE) (FDR < 0.01) and downregulated DE TEs rather than in upregulated DE TEs and non-differentially expressed TEs (FDR > 0.01). However, the mean expression of upregulated DE TEs is about 25% higher at 3 h and 100% higher at 9 h in comparison to background at 0 h, while non-DE TEs remained steady across all measured time points (Fig. 1B). The mean expression changes of host upregulated DE TEs highly correlates to IAV replication (Fig. 1B, Suppl Fig. 1A). In contrast to transposons, the mean expression level of host genes remained relatively steady for both total genes and DE genes. Although the expression level of upregulated genes jumped to 50% and 20% at the 3 and 6 h samples respectively, the change does not temporally correlate to virus replication dynamics through all time points (Suppl. Fig. 1B). These results suggest that the host transposon expression is likely influenced by early IAV infection and reflects the dynamics of virus replication.

We further analyzed and plotted the top differentially expressed TE families. The same TE family or subfamily can be presented in both up and down-regulated categories due to the nature of individual TE families spreading over hundreds or thousands of different genomic locations (Fig. 1C). In general, we have observed that up-regulated TE families (e.g. Alu, SVA, ERV1, L1, etc.) are heavily influenced by IAV at different infection time points (Fig. 1D). Many down-regulated TE families are also impacted by virus at early time points, but lack a clear temporal relationship to IAV replication (Suppl Fig. 1C). These results demonstrate that although both genes and TE are subjected to IAV infection, differentially expressed TEs, in particular upregulated TEs, are likely more sensitive to IAV replication.

### A group of virus-induced-TEs overlap the peak regions of chromatin remodeler CHD4, histone marker H3K27ac, and H3K9me3

**Figure 2:**
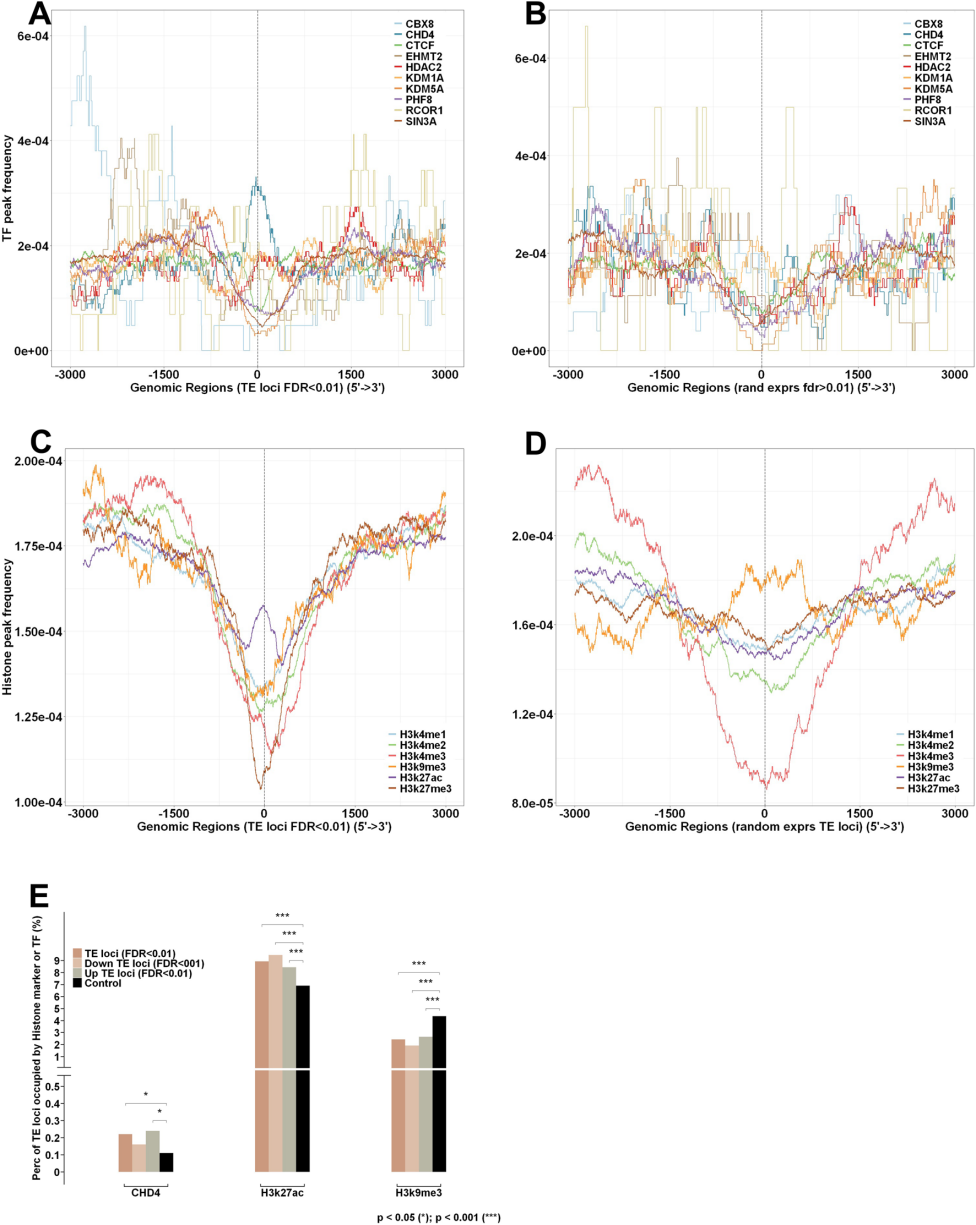
The spatial relationship between the DE TE loci and CHD4 binding sites, H3k27ac and H3k9me3 abundant regions. (**A**) CHD4 binding sites are more enriched in the DE TE regions (FDR < 0.01) than other 9 remodelers; in contrast to (**B**) non-DE TE regions (FDR > 0.01). (**C**) H3k27ac is abundant in the DE TE loci. (**D**). H3k9me3 is abundant in the non-DE TE loci. (**E**). Chi-square test demonstrated that the enrichment of CHD4, H3k27ac and H3k9me3 in the DE TE regions is statistically significant, which random selected TE loci (black) is control.

The ENCODE database contains many high-quality datasets for us to study the spatial relationship between TE transcripts and histone markers as well as TE transcripts and chromatin remodelers^10^. Although the histone modification data was not available for IAV infected A549 cells at the time we conducted this study, data from non-infected A549 cells provided a good foundation to measure the genomic spatial relationship of virus-induced-TEs. After exploring the ENCODE data matrix^10^, we exploited 6 histone marker and 10 chromatin remodeler datasets for a genome-wide spatial relationship analysis of virus-induced-TEs. All ENCODE datasets were derived from control experiments in A549 cells without treatment, which is comparable to the mock samples of IAV time series data. The name and label of the ENCODE datasets used in this analysis are listed in Supplemental Table 1. We first plotted the genome-wide occupancy for the 10 chromatin remodelers in the virus-induced-TE regions (FDR < 0.01). 12,000 randomly selected non-DE TE (FDR > 0.01) were used as the control. Of the 10 chromatin remodelers, we found that only CHD4 has a higher binding ratio in the virus-induced-TE regions as compared to non-DE TEs (Fig. 2A,B). The CHD4 binding sites tend to be in the up-regulated DE TE regions rather than in down-regulated TE regions (Suppl. Fig. 2A,B). With the most rigorous overlapping criteria (0 bp gap allowed in between two regions), we quantitatively measured the overlap between 2825 CHD4 peaks and 21,349 DE TEs. The result demonstrated that about 0.2% (48/21,349) of virus-induced-TEs are completely overlapping CHD4 peak regions, which is significant by Chi-square test in comparison to randomly selected non-DE TE loci (13/12,000) (Fig. 2E, left panel). However, a majority of virus-induced TEs (32/48) derived from CHD4 binding regions are upregulated (Fig. 2E, left panel). CHD4 is a nucleosome remodeler in the Snf2 family and plays an important role in epigenetic transcriptional repression^18^. Although only a small fraction of virus-induced-TEs (mostly upregulated TEs) were derived from CHD4 binding regions, this suggests that TE transcriptional repression by CHD4 was removed from these regions, which further indicates that the chromatin status changes from repressive to permissive in these genome regions to promote the TE transcription during IAV infection.

Of the 6 ENCODE histone markers, we found that H3K27ac has a higher occupancy in the virus-induced-TE regions in non-infected A549 cells (Fig. 2C), while H3K9me3 is more abundant in the randomly selected non-DE TE regions (Fig. 2D) meaning chromatin configuration is more repressive and less accessible in these regions in comparison to DE TEs. The H3K27ac occupancy in the DE TE regions shows no difference between upregulated and downregulated TEs (Suppl Fig. 2C,D). Using a rigorous overlapping strategy, we were able to identify 1907 virus-induced-TEs that are derived from H3K27ac abundant regions, which is about 8.9% of overall virus-induced-TEs and significantly higher than the 6.9% (828) of non-DE TEs (Fig. 2E, middle panel). We also uncovered 515 virus-induced-TEs that are occupied by H3K9me3, which is 2.4% of overall DE TEs and significantly lower in comparison to the 4.3% (522) of non-DE TE loci (Fig. 2E, right panel). These results demonstrate that about 9% of virus-induced-TEs are derived from H3K27ac abundant regions and 2.4% from H3K9me3 peak regions. The higher percentage of non-DE TEs that overlap H3K9me3 peak regions suggests that more TEs are suppressed by H3K9me3 in the genome prior to virus infection in comparison to post-infection.

H3K27ac is thought to be an epigenetic marker for enhancers in mammals, and its modification reflects cell type specific gene expression^19,20^. A recent study has shown that the enhancer activity cannot be determined by H3K27ac alone^21^. These findings suggest that H3K27ac likely works with other chromatin proteins to maintain poised chromatin status in its abundant regions. In our analysis, of the 2825 overall CHD4 binding sites, 2465 overlap with H3K27ac peaks, which indicates the majority (87%) of CHD4 binding sites are also occupied by H3K27ac (Suppl Fig. 2E). By comparing concurrency of CHD4 and H3K27ac in virus-induced-TEs to the non-DE TE control, we found that such concurrency only exists in virus-induced-TEs (Suppl Fig. 2E,F). High concurrency of CHD4 and H3K27ac in the virus-induced-TE regions may suggest functional dependency between CHD4 and H3K27ac, where CHD4 reduces chromatin accessibility and suppresses transcription events to maintain poised enhancer status prior to virus infection. Since CHD4 associated DE TEs are only a small fraction of overall virus-induced-TEs, this suggests that the rest of H3K27ac enhancers may rely on a different chromatin remodeler or mechanism to maintain poised genomic status in uninfected host cells. Of the total virus-induced-TEs, about 2.4% are derived from H3K9me3 genome regions, which is significantly lower than the 4.3% derived from randomly selected expressors. The number of virus-induced-TEs from H3K9me3 regions is quite surprising because H3K9me3 is thought to be the chromatin suppressive marker and the transcription activities in the genomic regions with abundant H3K9me3 should be very low. However, recent studies have shown that H3K9me3 is also present in poised enhancers^22^, which is likely related to cell type specific control^23^, though the mechanism is unclear. Our results support other groups’ discoveries and indicate that a small fraction of virus-induced-TEs are likely derived from poised enhancers where H3K9me3 is abundant.

### A proportion of virus-induced-TEs are very likely host eRNAs or part of eRNAs

**Figure 3:**
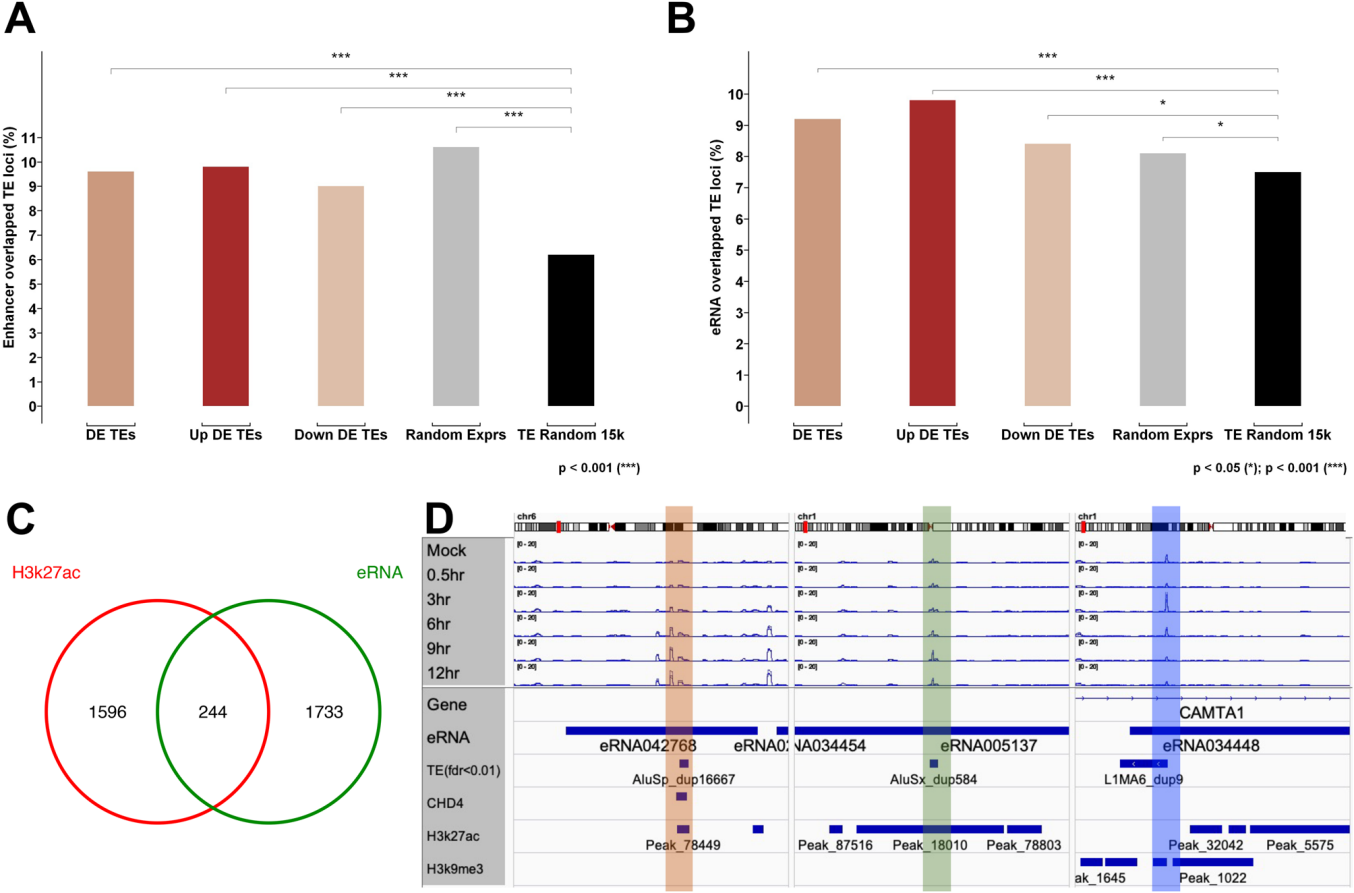
The spatial relationship between DE TEs and the predicted enhancers or eRNAs. (**A**) the DE TEs are significantly overrepresented in the predicted distal enhancer regions in contrast to random selected TEs (black), chi-square test. (**B**) using published eRNA dataset for verification, we found that the DE TEs are significantly overrepresented in eRNA regions, random selected TEs is the test control, chi-square test. (**C**) the 244 DE TEs that derived from H3K27ac occupied regions also overlap the eRNAs (*p* < 0.05, hyper geometric test). (**D**) IGV displays the expression alteration of eRNA related DE TEs in the regions of CHD4 and H3k27ac enriched (left), H3k27ac alone (middle) and H3k9me3 alone (right).

Analysis of the genomic spatial relationship between virus-induced-TEs and epigenetic markers led to a discovery of a connection between DE TEs and enhancers. In addition, as a verification process, we also compared virus-induced-TEs to the computationally predicted enhancers of the human genome^11^. The results with predicted enhancers confirm what we observed from the spatial relationship analysis, in which about 9–10% of IAV induced DE TEs are derived from the predicted enhancer regions (Fig. 3A). Moreover, applying the same algorithm to a SARS-CoV-2 dataset^13^ and a dataset of multiple virus studies^14^ yielded very similar results (Suppl Fig. 3A). The results from different datasets and analysis methods suggest that a proportion of virus-induced-TEs are from enhancer regions of host cells. Combining both computationally predictedt results and spatial relationship analysis, we summarized that ~ 9% of virus-induced-TEs were derived from H3K27ac abundant genome regions (k27-like enhancers) and 2.4% from H3K9me3 regions (k9-like enhancers). Of the k27-like enhancers, ~ 2% of them are concurrent with CHD4. However, the spatial relationship between virus-induced-TEs and enhancers does not necessarily indicate physiological relevance to host gene expression regulation.

To address this question, we designed another experiment to examine whether virus-induced-TEs overlap human eRNAs or parts of eRNAs, because eRNAs are thought to play regulatory roles in downstream gene expression regulation^15^^,^^16^^,^^17^^,^^24^. We adopted a recently published eRNA dataset^9^ for a detailed analysis of virus-induced-TEs and known eRNAs in both a spatial and orientation manner. To analyze the spatial relationship between virus-induced-TEs and eRNAs, we created three testing datasets; overall virus-induced-TEs (FDR < 0.01), upregulated DE TEs, and downregulated DE TEs (FDR < 0.01). We also generated two control datasets; a random selection of 12,000 non-DE TEs (Rand_exprs, FDR > 0.01), and a random selection of 15,000 TEs from the entire TE pool regardless of expression (TE_rand15k, more than 95% has no detectable transcripts). Each of the test and control datasets overlap the known human eRNAs during analysis, obtaining a percentage of eRNA overlapped TEs, respectively. By comparing the eRNA overlapped TEs of test datasets to controls, we may determine whether the virus-induced-TEs are prone to eRNAs (Fig. 3B, Chi-sq test). Using randomly selected non-DE TEs as a control, we found that only overall DE TEs (9.2%) and upregulated DE TEs (9.8%) are significant (Suppl. Fig. 3C). These results demonstrate that a significant amount of upregulated DE TEs (9.8%) overlap the eRNAs in comparison to both randomly selected non-DE TEs (8.1%) and randomly selected TEs (7.5%). However, downregulated DE TEs (8.4%) demonstrate no significant difference from non-DE TEs. These results indicate that a significant amount of virus-induced-TEs, particularly those that are upregulated, are derived from the same enhancer regions where the eRNA transcription may take place.

Of the total 1977 eRNA overlapped DE TEs (TE-eRNAs), 244 overlapped the DE TEs that are derived from H3K27ac regions (hypergeometric test, *p* < 0.001) (Fig. 3C). The result shows partial but not complete overlap, which may reflect that the eRNA dataset originated from active enhancers of entire human tissues and cells, while virus-induced-TEs are likely derived from poised enhancers of A549 cells.

To have a direct observation of TE-eRNAs, we loaded sequencing data of IAV infected samples together with the eRNA data and epigenetic markers CHD4, H3K27ac or H3K9me3 into the IGV genome browser. We observed TE AluSp_dup16667 expression upregulated in the 3, 6, 9, and 12 h samples (Fig. 3D, left panel, highlighted in orange), where CHD4 binding sites and H3K27ac peak are concurrent. In the middle panel (highlighted in green) of Fig. 3D, TE AluSx_dup584 is upregulated in 6, 9 and 12 h samples, where the H3K27ac peak is present. Both AluSp_dup16667 and AluSx_dup584 are autonomous TEs in the intergenic genome region. The TE L1MA6_dup9 is upregulated in 3 h samples, but downregulated in 9 and 12 h samples (Fig. 3D, right panel, highlighted in blue), whereas the intragenic region of CAMTA1 is occupied by H3K9me3. In addition to TE-eRNAs, we also observed an intergenic DE TE region where eRNA is not reported, but CHD4 and H3K27ac are concurrent (Suppl Fig. 3C). This piece of data may unveil an unknown enhancer region and potential TE-eRNAs. The full lists of DE TEs and the concurrent epigenetic markers are in Supplemental Tables 2, 3 and 4. Taken together, these results also suggest that the virus-induced-TEs may play very complex regulatory roles in host gene expression during virus infection.

### TE-eRNA targets are enriched in the upregulated DE genes of IAV infected samples

**Figure 4:**
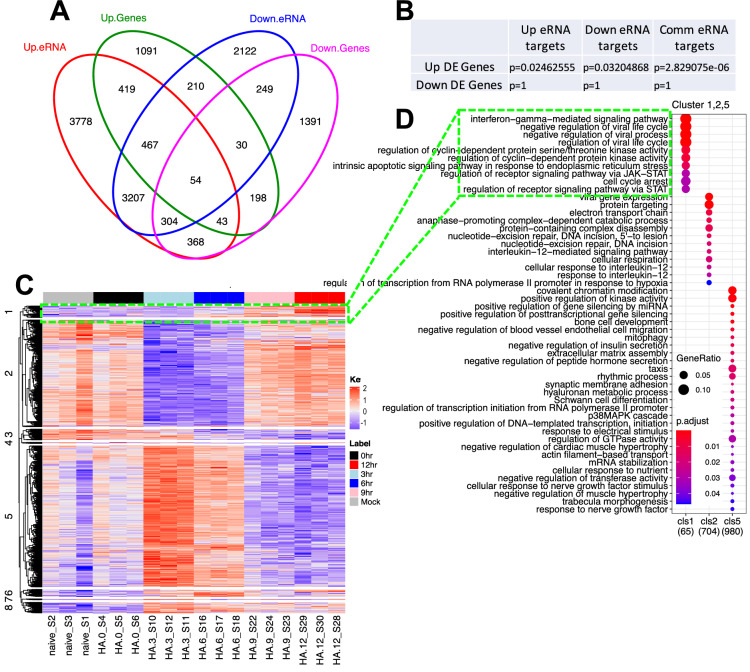
The DE TE associated eRNA targets are significantly enriched in up regulated DE genes. (**A**) Venn diagram shows the overlap between up or down DE genes and up or down DE TE related eRNA targets. The hypergeometric tests were performed, and the results are presented in B. (**B**) Hypergeometric test shows DE TE related eRNA targets are significantly enriched in the upregulated DE genes, e.g. the subset of common eRNA targets (intersection marked 3207,467,304,54) overlaps the subset of upregulated genes (intersection marked 467,54). (**C**) Hierarchical clustering analysis for the overlapped 2144 DE genes. (**D**) GO term enrichment analysis results demonstrate the IFN and virus life cycle pathways in the cluster 1.

If eRNA reflects enhancer transcription activity, eRNA targeted gene expression will represent functional connection between enhancer and host genes. In this sense, whether the TE-eRNA targets are differentially expressed or not in the IAV infected samples will be critical to inferring the potential regulatory role of virus-induced-TEs. The eRNA dataset containing eRNA targeted genes allows us to test this hypothesis. We first queried the eRNA targeted gene dataset with TE-eRNAs, down- and up-regulated TE-eRNAs, respectively, which resulted in 11,251, 6643 and 8640 TE-eRNA targets (named as TE-eRNA targets, down- and up-TE-eRNA targets). These TE-eRNA targets were compared with differentially expressed genes (DE genes) of IAV time series samples. The hypergeometric test was subsequently applied for the overrepresentation significance assessment. We found that both down and up-TE-eRNA targets are enriched in the upregulated DE genes (*p* < 0.05, Fig. 4A,B). The results suggest that either down- or up-regulated virus-induced-TEs tend to upregulate their target genes during IAV infection.

By overlapping TE-eRNA targets with 4824 overall DE genes, we obtained 2144 DE genes (eRNA DE genes). Applying hierarchical clustering and GO TERM analysis for eRNA DE genes, we found that the IFN pathway, virus defense and JAK-STAT pathway genes are enriched in cluster 1, where the expression level increases as virus replication progresses (Fig. 4C,D). The enrichment of the IFN pathway in the eRNA DE genes suggests that TE-eRNAs are likely an important regulatory path during IAV infection. The detailed pathway information from eRNA DE genes is available in Supplemental Table 5. The overrepresentation of TE-eRNA target genes in the DE genes suggests the potential regulatory roles of virus-induced-TEs.

From the above results (Fig. 2E), we have revealed that virus-induced-TEs can be derived from either H3K27ac or H3K9me3 abundant enhancers, where a proportion of H3K27ac pre-occupied enhancers are concurrent with CHD4. Taking this into account, we categorized TE-eRNAs into three groups, H3K27ac, H3K27ac plus CHD4 and H3K9me3. We interrogated whether the three groups of TE-eRNA targets are all impacted by IAV infection. In this analysis, TE-eRNAs overlapped CHD4 binding, H3K27ac or H3K9me3 peak regions with a slightly relaxed gap size of maximum 500 bp instead of 0 bp, in which we expect to include more genes in the analysis. We found about 2477 eRNA targets also present in the 4824 DE genes (2477 is bigger than the above 2144 due to a relaxed gap size). Of 2477 eRNA targets, 210 relate to CHD4 binding region, 2250 to H3K27ac and 707 to H3K9me3 (Suppl Fig. 4A). These data suggest that IAV likely impacts all three types of enhancers.

In summary, our results demonstrate the following: (1) the host upregulated TEs temporally correlate with virus replication, (2) a significant number of IAV induced DE TEs are derived from host enhancer regions; the spatial relationship between DE TEs and enhancers also exists in SARS-CoV-2, RSV and HPIV3 infected samples, suggesting such a genomic relationship between virus-induced-TEs and enhancers is likely common in RNA virus-host interaction, (3) the IAV-induced-TEs are either eRNAs or part of eRNAs, which originate from the enhancers with three different epigenetic modifications, and (4) the TE-eRNA genes are significantly overrepresented in the DE genes of IAV infection time series samples, which indicates that during IAV infection the host DE genes or a proportion of DE genes are likely influenced through TE related enhancers and eRNA pathways. Our results suggest that IAV likely interacts with host cells by changing the chromatin accessibility from repressive to permissive in the enhancer regions, regulating host downstream gene expression, where TEs are docked and likely play important regulatory roles.

## Discussion

For a long period of time, transposon element expression and its potential influences on gene expression regulation have not been deeply studied. Our previous work revealed that the virus induced transposon expression in human and mouse^1^ and another group also found virus induced transposon expression in *Drosophila* somatic tissues^2^. These results from independent studies suggest that viral impact on host transposon expression is likely a common phenomenon across many organisms and viral species. However, many virus induced transposon transcripts are influenced by gene expression due to intron retention and gene readthrough^25–27^. In humans, only about 9–10% of transposon transcripts are from intergenic genome regions^1^, which makes study of this phenomenon even more challenging due to interference of gene transcripts. Even though TE transcription is real, many questions still remain, e.g. what function TE transcripts have, and what roles they might be playing besides canonical transpositions. Therefore, further characterization of virus induced transposon transcripts is necessary to fully understand their functionality.

**Figure 5:**
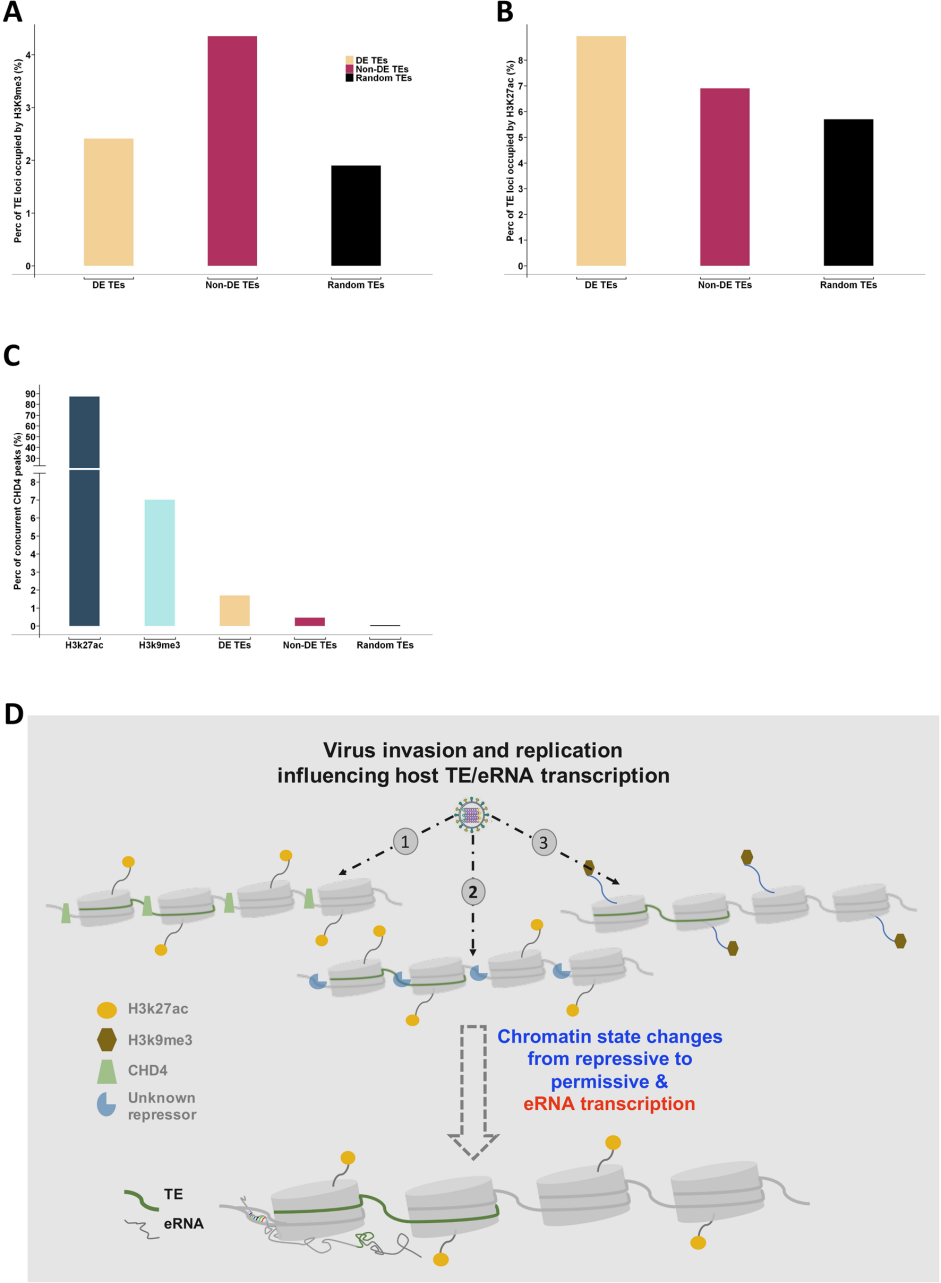
Chromatin accessibility in transposon regions in uninfected A549 cells. (**A**) the number of H3k9me3 occupied non-DE TEs is about twice higher than DE TEs, which suggest IAV likely impacts on TE regions with less H3k9me3 occupancy. (**B**) the DE TEs tends to be derived from H3k27ac occupied TE regions in contrast to non-DE TEs and random controls. (**C**) majority of CHD4 binding regions are H3k27ac occupied. (**D**) A putative model illustrating how virus may interact with the host to change chromatin status, leading to activation of poised or silent enhancers.

The risk of active TE transcription is TE transposition and chromosomal rearrangements, which may cause inheritable diseases in humans^28,29^. Therefore, TE activity, in particular canonical activity, is in general repressed in the human genome by multiple mechanisms of chromatin condensation, epigenetic transcriptional silencing, and targeting by small interfering RNAs^30^. Investigating the epigenetic modifications in non-infected cells for the TE regions can build a foundation for understanding virus-induced-TEs. We selected six histone markers and ten chromatin remodelers from the ENCODE data matrix, which represent permissive and repressive chromatin status in the A549 cell line that was also used for the IAV infection time series study. Surprisingly, about 4.3% of non-differentially expressed transposons are derived from H3K9me3 occupied regions; however, only about 1.9% of randomly selected transposon genomes widely overlap H3K9me3 occupied regions (Fig. 5A). Low occupancy of H3K9me3 in the randomly selected TEs is somewhat understandable as the vast majority of repetitive DNAs are part of the gene reading frame or intron regions. What’s surprising is significantly higher H3K9me3 occupancy in non-differentially expressed transposon regions (4.3%) compared to differentially expressed TE regions (2.4%) (Fig. 5A). This result suggests that H3K9me3 in these regions is likely not just a repressive marker^22,23^, but also a landmark of poised chromatin regions for the external signals or stimuli, which resulted in expression changes for 2.4% TEs during IAV infection.

H3K27ac is thought to be an epigenetic marker that distinguishes active enhancers from poised ones^20^. In our study, H3K27ac is found abundant in the DE TE regions of both up- and down-regulation (Fig. 2C, Suppl Fig. 2C,D), where H3K4me1 occupancy is very low, suggesting that H3K27ac occupied TE regions are poised and less accessible to transcription events in non-infected host cells. Quantitatively, the percentage of H3K27ac overlapped DE TEs over total DE TEs is about 8.9%, which is statistically significant in comparison to non-DE TEs (6.9%) and randomly selected TEs (5.7%) (Fig. 5B). This indicates H3K27ac abundant TEs likely switched from poised to active and were differentially transcribed during IAV infection. It is unclear how H3K27ac enriched TEs remained poised before infection and turned into permissive during infection. However, the genome loci concurrency between CHD4 and H3K27ac likely solves this puzzle, where the system uses CHD4 to repress transcription in the H3K27ac occupied enhancer regions. Although 87% of CHD4 binds to H3K27ac peak regions, only 1.7% of virus-induced-TEs are CHD4 and H3K27ac concurrent (Fig. 5C), indicating that some other DNA binding proteins play similar roles to suppress TE/enhancer expression in the non-infected host cells. Based on our study, we proposed a putative virus and host interaction model where the virus activates host enhancers by changing chromatin accessibility, with TEs playing as either *cis* and/or *trans* elements in regulating neighboring or distal genes. (Fig. 5D).

eRNAs are the product of enhancer transcription and play critical roles in regulating gene expression^15^^,^^16^. The virus-induced-TEs significantly overlapping with eRNA provides direct evidence of enhancer activation and transcription during IAV infection (Fig. 3B). We also reported that a significant number of TE-eRNA targets are overrepresented in the DE gene (Fig. 4A,B). Of a total 4824 DE genes, 2477 (51.3% with relaxed gap size) overlap the TE-eRNA targets (Suppl Table 5). M any of those were also reported in earlier studies^12,31–33^, suggesting our results are supported by previously published discoveries. The genes that constitute the IFN mediated pathway and other innate immune gene pathways such as STAT signaling pathway are upregulated in the time series samples (Fig. 4C,D), which indicates that these genes are either suppressed or silenced in the host cells prior to IAV infection possibly through the poised enhancer mechanism. Our findings suggest that the poised enhancers and eRNAs where TE docked are likely involved in the gene expression regulation during the host defensive response to IAV infection, which are perhaps the missing pieces in the IAV and host interaction model suggested by peers^34–36^. Of the 4824 DE genes, 2347 do not overlap the TE-eRNA targets. This may be due to the fact that the eRNA target list obtained from the most recent updates is still incomplete, which suggests more computational work to be done in the future. It is also possible that the rest of the DE genes are regulated by different mechanisms. In summary, our study for the first time has revealed a possible new role of TE transcription in regulating downstream gene expression through the poised enhancer and eRNA mechanism during IAV infection. These interesting results may also lead to a new method development for precisely probing enhancers and their targeted genes, in addition to new insights for virus-host interaction mechanisms and therapeutic approaches.

## Materials and methods

### RNA sequencing data acquisition and processing, differentially Gene and TE analysis

All the data in this study was obtained from the GEO database (GSE147832, GSE147507)^12,14^ and NGDC database (CRA002606)^13^. The fastq files were first mapped to human genome assembly (Homo.sapiens.GRCh38) using bowtie2 aligner (v.2.3.4)^37^ with parameters (bowtie2 -local -L 22 -N 1 -k 100). After sorting with samtools (v.1.9)^38^, the mapped reads were used to summarize genes (Homo_sapiens.GRCh38.87.gtf) and TEs (GRCh38_Ensembl_rmsk_TE.gtf) using featureCounts (v.1.5.1)^39^ with either strand parameter on (reverse) for projects GSE147832 and GSE147507, or strand parameter off for the project CRA002606 due to non-strand specific protocol applied in the sequencing. The outputs were subsequently used for statistical assessment for the differentially expressed genes and TEs by R/bioconductor package (version 4.0.3/3.12) edgeR (v.3.32.1)^40,41^. The cutoff for the genes and TEs is FDR < 0.01 with one exception of FDR less than 0.001 for Cov2 24 h sample of CRA002606 dataset.

### Virus reads mapping and processing

The raw fastq files of IAV infected samples were mapped to in-house bowtie2 index of influenza A virus (bowtie2 -local -L 22 -N 1 -x). The bowtie2 index file along with the fasta sequences and annotation files for IAV and SARS-CoV-2 viruses are available upon request. The featureCounts was used for virus reads assignment. The count per million (CPM) was introduced for normalization of virus reads crossing time series samples, in which the virus reads were normalized against overall library reads (mapped virus reads + host gene reads) per sample for IAV dataset.

#### ENCODE functional genomic datasets and spatial relationship to DE TEs

ENCODE functional genomic data files^10^ were used for this study as listed in Supplemental Table 1. The R/Bioconductor package ChIPseeker (v.1.26.2)^42^ was used for generating epigenetic marker distribution around DE TE and control TE regions. To create genomic regions for plotting, each TE was extended from the center of the TE to both up and down stream for 3000 bps. The peak file of epigenomic markers was used for counting peak frequency in the TE regions with default parameters of ChIPseeker.

To screen the DE TEs that overlap epigenetic markers (CHD4, H3K27ac, H3K9me3), we used bedtools (v.3.5) (bedtools closest -a TEs.bed -b Enhancers.bed -mdb all -D b) for measuring the distance of two genomic features^43^. The 0 bp gap between DE TEs and epigenetic markers was used for determining the spatial relationship of DE TEs.

The randomly selected 10,000 TE expressors (non-DE TE, FDR > 0.01) were used as the control for either plotting and/or statistical testing.

### Distal enhancer dataset and DE TE overlapping

The distal enhancer dataset was downloaded from the Enhancer Atlas website (version 2.0)^11^. The enhancer coordinates were updated to genome version HG38 with UCSC liftover utility before further analysis^44^. The distal enhancer data was then overlapped with DE TE loci using bedtools (v.3.5) (bedtools closest -a TEs.bed -b Enhancers.bed -mdb all -D b). To calculate the percentage of distal enhancer related (overlapped) DE TE loci, the number of distal enhancers overlapped DE TE loci were divided by total number of DE TE loci in the same category or same time point. The randomly selected 15,000 TEs are the controls for plotting and statistical testing. Of 15,000 randomly selected TEs, less than 300 have expression values bigger than 0. In addition to the randomly selected TEs, we also used a non-DE TE as a control. The difference between the randomly selected TEs and non-DE TE control is that non-DE TEs have the detectable expression values.

### Enhancer RNA and DE TE overlapping

The enhancer RNA dataset of whole human tissues was downloaded from HeRA atlas^9^. The enhancer RNA coordinates were updated to genome version HG38 with UCSC liftover utility before further analysis. To generate the distance matrix, bedtools (v.3.5) (bedtools closest -a TEs.bed -b Enhancers.bed -mdb all -D b) was used for calculating the distance between two genomic features. The randomly selected TEs and non-DE TEs were used as controls for this study to assess the statistical significance. To screen TE eRNAs, we allowed only 0 bp gap between two features.

To obtain TE eRNA target genes, we downloaded eRNA targets from HeRA atlas. We grouped TE eRNA targets into overall DE TE, up DE TE, or down DE TE related and plotted a venn diagram for visualizing the TE eRNA targets and differentially expressed genes (DE genes). The 2144 genes that are common between TE eRNA targets and DE genes were further used for hierarchical clustering analysis.

### Gene clustering, GO TERM analysis, IGV genome browser and statistical test

The ComplexHeatmap R/bioconductor package (v.2.6.2)^45^ was used for gene clustering and heatmap visualization. For the GO TERM analysis, we used clusterprofiler R/bioconductor package (v.3.18.1)^46^. We used IGV genome browser for navigating and visualizing the individual eRNA related DE TEs and their spatial relationship to epigenetic markers. The R function chisq.test() and phyper() were used for either chi-square or hypergeometric test for the statistical significance assessment in our study.

## Datasets used in this study

The RNAseq data were downloaded from GEO database (GSE147832, GSE147507)^12,14^ and NGDC database (CRA002606)^13^. The Chipseq data were downloaded from ENCODE database and dataset names are listed in Supplement Table 1.

## Supplementary Information

Supplementary Information 1.

Supplementary Information 2.

Supplementary Information 3.

Supplementary Information 4.

Supplementary Information 5.

Supplementary Information 6.
