# Supplementary material for: Characterization of influenza A virus induced transposons reveals a subgroup of transposons likely possessing the regulatory role as eRNAs
Source: Sci Rep. 2022 Feb 9;12:2188. doi: 10.1038/s41598-022-06196-6 (PMC8828846; doi:10.1038/s41598-022-06196-6)
Supplement: Supplementary file 6 — Supplementary Information 6. [file 41598_2022_6196_MOESM6_ESM.docx]

Characterization of influenza A virus induced transposons reveals a subgroup of transposons likely possessing the regulatory role as eRNAs

Steven S. Shen^1,3^*, Hezkiel Nanda^1^, Constantin Aliferis^1^, Ryan A. Langlois^2^

**Affiliations:**

^1^ Institute for Health Informatics, University of Minnesota, Minneapolis, MN, 55455.

^2^ Center for Immunology, Department of Microbiology and Immunology, University of Minnesota, Minneapolis, MN, 55455.

^3^ Clinical Translational Science Institute, University of Minnesota, Minneapolis, MN, 55455

Supplementary Materials:

1. Supplement Figures S1-S4
2. Supplement Table S1: List of the datasets from ENCODE.

Legend: the accession numbers of ENCODE datasets and their corresponding descriptions that used for this study are listed in the supplement table 1.

1. Supplement Table S2-4: the results of the virus induced TEs overlapping histone markers and chromatin remodeler.

Legend:

1. Table S2: the overlap of CHD4.
2. Table S3: the overlap of the H3K27ac.
3. Table S4: the overlap of the H3K9me3.
4. Supplement Table S5: **the complete GO TERM results of the hierarchical clusters in Figure 4C.**

Legend: the detailed GO TERM list of 2144 DE genes that overlap TE eRNA targets that includes IFN alpha and gamma pathways, and other virus defensive pathways.
